# Multi-country investigation of factors influencing breeding decisions by smallholder dairy farmers in sub-Saharan Africa

**DOI:** 10.1007/s11250-018-1703-7

**Published:** 2018-09-12

**Authors:** G. Mwanga, F. D. N. Mujibi, Z. O. Yonah, M. G. G. Chagunda

**Affiliations:** 1 The Nelson Mandela African Institution of Science and Technology, Arusha, Tanzania; 2 Usomi Limited, Suite 13R, Hardy Post, Ushirika Road, Karen, Nairobi Kenya; 3 Department of Animal Breeding and Husbandry in the Tropics and Subtropics, University of Hohenheim, Garbenstr.17, 70599 Stuttgart, Germany

**Keywords:** Artificial insemination, Breeding decisions, Bull mating, Dairy farming, Small-scale farmers

## Abstract

Artificial insemination (AI) and selective bull mating are considered as robust methods for dairy cattle breeding. Globally, these methods have been used to enhance productivity and realize rapid genetic gains. However, these technologies have had low adoption rates in sub-Saharan Africa (SSA). Even though available evidence suggests that this is due to various infrastructural and technical challenges. There is limited information about what drives this low uptake of AI from a farmer’s perspective. Therefore, the main objective of this study was to determine and characterize factors that influence the choice by smallholder farmers between bull service and AI for dairy cow breeding. Further, the relationships between the breeding choices and the bio-physical elements of dairy farming, mainly, farmer characteristics, household income levels, farm management practices, and institutional support structures, were investigated. Data were collected through face-to-face interviews from a total of 16,308 small-scale dairy farmers in Ethiopia (*n* = 4679), Kenya (*n* = 5278), Tanzania (*n* = 3500), and Uganda (*n* = 2851). The questionnaire was coded in an electronic form using Open Data Kit (ODK) platform to allow for real-time data entry and management. Descriptive statistics, chi-square test, and a t-test were used to evaluate the independent and dependent variables, while logistic regression and factor analysis were used to identify factors that influenced farmers’ breeding decisions. Results showed that there was a significant difference in animal husbandry practices between farmers who used artificial insemination (AI) and those who practiced bull mating. The majority of farmers who used AI kept records, purchased more animal feeds, had more labor by hiring workers whose average wages were higher than those of bull service farmers. However, farmers who used AI pay more for services such as water access and breeding while their service providers had to cover long distances compared to farmers who used bulls. This indicates limited access to services and service providers for AI farmers. The ratio of AI to bull service users was even for Ethiopia and Kenya, while in Uganda and Tanzania, more farmers preferred bull service to AI. It was established that the factors that influence farmers’ breeding decision were not the same across the region. Factors such as farmer’s experience in dairy farming, influence of the neighbor, farmer’s ability to keep records, and management practices such as water provision and availability of feeds had a significant association (*p* < 0.001) with AI adoption among dairy farmers. In contrast, large herd and large land size negatively influenced AI adoption. Institutional settings including cost of AI service and the distance covered by the service provider negatively affected (*p* < 0.001) the choice of AI as a breeding option. There was a high probability of continued use of a specific breeding method when there was a previous conception success with that same method. Based on the results obtained, we recommend that improvement of institutional settings such as the availability of AI service providers, as well as better access to services such as water, animal feed, and animal health provision, be treated as critical components to focus on for enhanced AI adoption. Most importantly, there is a need to avail training opportunities to equip farmers with the necessary skills for best farm management practices such as record keeping, proper feeding, and selective breeding.

## Introduction

Dairy farming is an important source of income and nutrition for many smallholder farmers in Eastern Africa. Generally, in sub-Saharan Africa (SSA), cow milk production is predominant in Eastern Africa which accounts for 68% of the continent’s cow milk output with Ethiopia, Kenya, and Tanzania being among the top dairy producers in Africa (Bingi and Tondel 2015). The major stimulator of the growth in smallholder dairying is the recent increase in demand for fresh milk and other value-added milk products triggered by a growing population (Gillespie and van den Bold 2017). This demand is replicated in all regions of sub-Saharan Africa (The World Bank 2014). However, per animal productivity is still low mainly because of using inappropriate genetics, poor husbandry practices, and feed scarcity. With the world population projection to hit 9.15 billion in 2050 (UNPD 2008), the unit productivity of the animals must be increased. As such, use of modern breeding technologies and best management practices for more effective production is of critical importance if this demand is to be met. Increasing milk yield per cow as opposed to the number of animals would be ideal for proper utilization of available feed resources. In light of the above, strategies to increase productive performance of animals must be in place (Morotti et al. 2016) specifically targeting small-scale farmers who make up majority of dairy farms in developing countries (Richards et al. 2015).

To enhance productivity and realized genetic gain, robust and practical germplasm delivery technologies and mechanisms such as artificial insemination (AI) and selective bull mating are fundamental. Since its introduction 60 years ago, AI has experienced a rapid diffusion and usage across the world due to its potential. The appeal for AI lies not only in its ease to obtain genetic improvement but also in its ability to eliminate costly venereal diseases and increase efficiency of bull usage, which decreases running costs (Lamb et al. 2016). Consequently, AI has been the most widely used reproductive technology in dairy farming and has been mainly adopted in developed countries and on commercial farms in developing countries.

Currently, AI is also well utilized in some African countries such as South Africa and Kenya (Omondi et al. 2017). However, the adoption rate in other SSA countries is still low especially among small-scale farmers (Tefera et al. 2014; Anthony et al. 2014). The reasons for the low uptake of AI by farmers have never been clearly established across the main dairying countries in Africa. Therefore, understanding the key drivers of a farmer’s choice for a particular breeding service is critical if the adoption rates are to be increased. This study sought to determine the following: (1) whether there were any observable differences in the usage of AI and bull service among smallholder farmers in the four SSA countries, (2) the factors that influenced a farmer’s decision on usage of AI or bull service, and (3) to investigate whether there were any differences on the main drivers either influencing or hindering a farmer’s decision-making with regard to breeding choices across the regions.

## Materials and methods

### Study sites

**Fig. 1:**
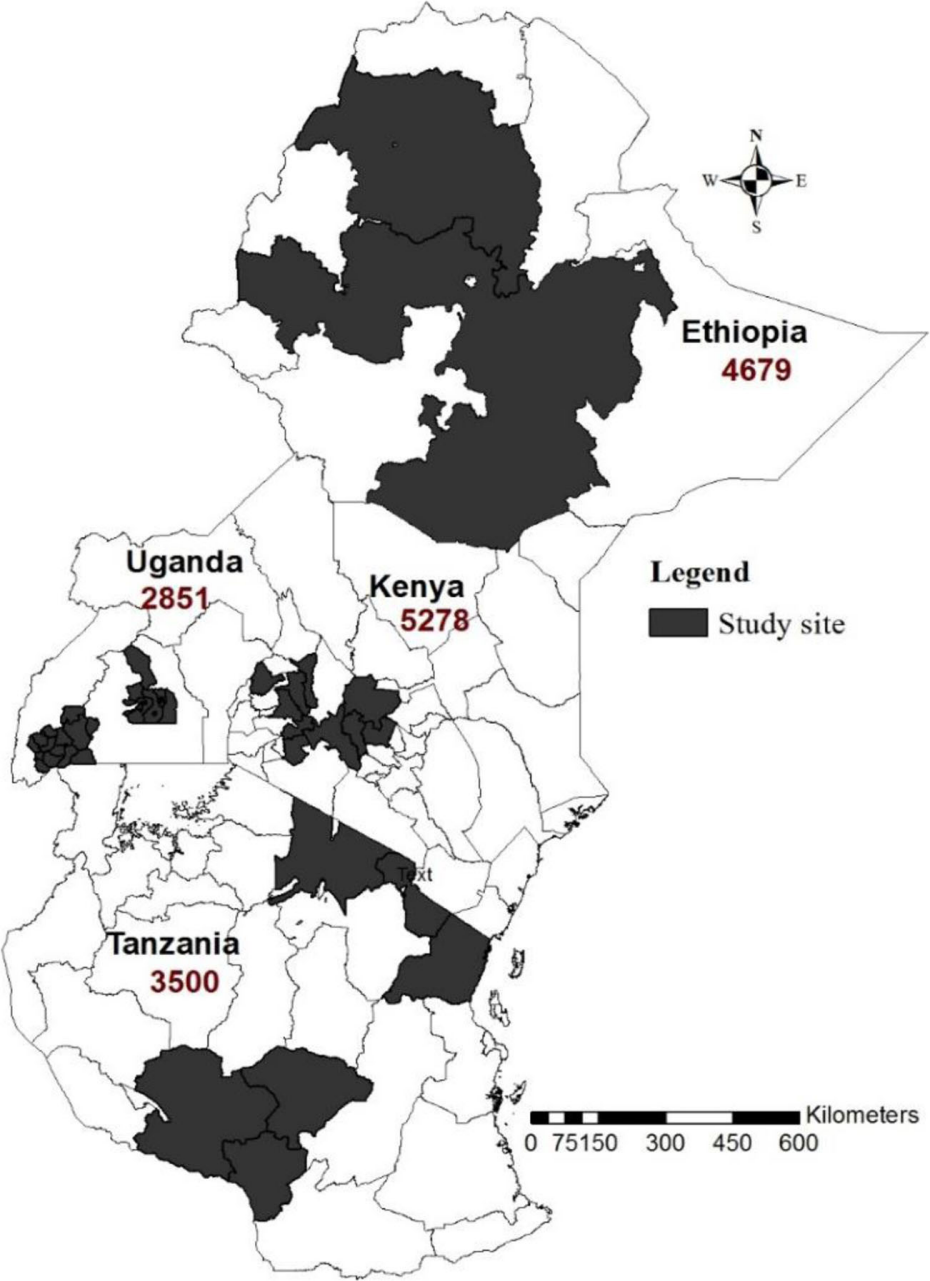
Map of the study regions and countries: Ethiopia, Kenya, Tanzania, and Uganda. The study focused on dairy farmers; hence, selection of study sites was done in traditional and emerging dairying zones

Data were collected in four countries: Ethiopia, Kenya, Tanzania, and Uganda. The study focused on dairy farmers; hence, study sites in traditional and emerging dairying zones were selected to maximize the number of dairy farmers to be included in the study. Figure 1 shows the study sites in the four surveyed countries. Four milk sheds were identified in Ethiopia: Addis Ababa, Asela, Bahir Dar, and Hawassa. In Kenya, three adjacent dairying zones were selected: Central, North Rift, and South Rift. In Uganda, three zones were selected based on their concentration of dairy activities. These were Kiruhura, Wakiso, and Mbarara. In Tanzania, six regions were selected: Arusha, Kilimanjaro, Tanga, Iringa, Njombe, and Mbeya.

A cross-sectional survey was conducted through face-to-face interviews of target farmers on their households over a 1-year period (from June 2015 to June 2016). A structured questionnaire coded in Open Data Kit (ODK) was used to capture data electronically. A total of 16,308 small-scale dairy farmers were interviewed as follows: Ethiopia, 4679; Kenya, 5278; Tanzania, 3500; and Uganda, 2851. After data cleaning and quality checking processes, only 13,095 respondents qualified for inclusion in the analysis and these were distributed as follows: Ethiopia, 2892; Kenya, 4400; Tanzania, 3236; and Uganda, 2555.

### Data and definition of variables

**Fig. 2:**
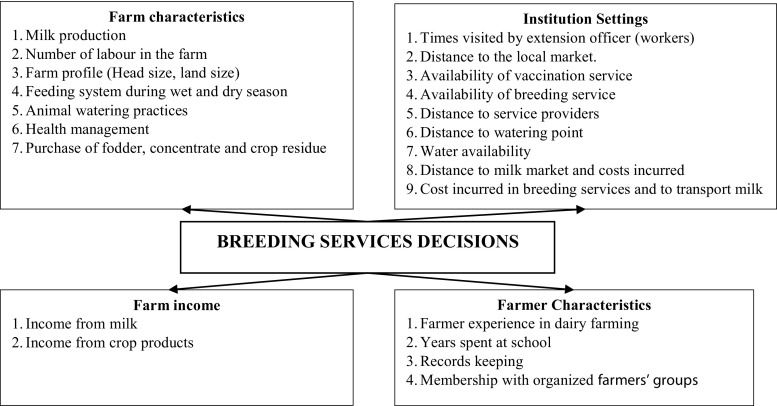
Decision framework on breeding choice between artificial insemination and traditional bull mating by dairy farmers. Four main characteristics of each business were included, i.e., farm characteristics, institutional settings, farm income, and farmer characteristics

Selection of the factors that influence choice of breeding method was based on the domain knowledge and empirical findings from literature. Figure 2 shows the factors that were tested and grouped into four components as explained below:

#### Farm characteristic variables

The following farm characteristic variables were analyzed: farm assets (land and herd size), total number of laborers in the household, and number of months a farmer purchased fodder, concentrate, and crop residues in the year preceding the survey. Data on animal production was limited to estimated milk yield at start, peak, and end of lactation for the best and worst animal in a herd. The values reported were average estimates based on farmer recall and not the actual realized yields. Two-factor scores were obtained after performing factor analysis on animal production variables including production at peak and lactation length for the best and worst animal. A score table (Table 1) was constructed by assigning different weights to several qualitative variables including binomial and other categorical measures based on their quantitative score.

**Table 1:** Weight allocations for categorical variables

	3. Household experience in dairy farming			4. Number of times used AI/Bull
1. Binomial variables Yes = 1, No = 0 2. Breeding methods Bull = 0, AI = 1	No experience = 0 1 to 5 years = 1 6 to 10 years = 2	11 to 15 years = 3 16 to 20 years = 4 21 to 25 years = 5	26 to 30 years = 6 30 to 40 years = 7 41 to 50 years = 8 More than 50 years = 9	Once = 1 Twice = 2 Three times = 3 Multiple = 4
5. Feeding System	6. Distance to market		7. Frequency of treating cattle diseases	
Mainly grazing = 1 Mainly stall feeding = 2 Only grazing = 3 Only stall feeding = 4 Transhumance all animals = 5 Transhumance some animals = 6	1 Km = 1 2 Km = 2 3 Km = 3 4 Km = 4	5 Km = 5 6 to 7 Km = 6.5 8 Km = 8 More than 8 Km = 9	Never = 0 Weekly = 1 a Every two weeks = 2 Monthly = 3	Every two months = 4 Every four months = 5 Twice a year = 6 More than a year = 7
8. Deworming times/year	9. Times vaccinated/year	10. Watering frequency/day		
Never = 0 Once = 1 Twice = 2 Three times = 3 Four times = 4 More than four times= 5	Once = 1 Twice = 2 Three times = 3 Four times = 4 Multiple = 5	None = 0 Once = 1 Twice = 2; Thrice = 3; Watering ad labium = 4		

#### Farmer characteristics

Evaluation of biographical variables included years of formal education and total number of children in the household. Dairy management variables included record keeping (1 = Yes, 0 = No) and methods for estrus detection. Other variables included membership to a farmer group (1 = Yes, 0 = No) and household experience in dairy farming (0 for no experience;1 for 1 to 5 years; 2 for 6 to 10 years; 3 for 11 to 15 years; 4 for 16 to 20 years; 5 for 21 to 25 years; 6 for 26 to 30 years; 7 for 30 to 40 years; 8 for 41 to 50 years; 9 for more than 50 years).

#### Infrastructural and institutional settings

Institutional setting variables included the following: number of times visited by an extension officer, distance to market in kilometers, availability of vaccination services (1 = Yes, 0 = No), availability of breeding services (1 = Yes, 0 = No), cost of breeding, distance to service provider in kilometers, availability of water (1 = Yes, 0 = No), distance to milk market in kilometers, and cost incurred to transport milk.

#### Farm income

Two variables, income from crop sales and income generated from milk sales, were included to represent sources of farm income.

### Model specification and data analysis

A multivariate logistic model was employed to identify factors that influence breeding decisions (usage or non-usage of AI). Two approaches were used in the data analysis. In the first approach, t-test and chi-square test were used to evaluate whether there was a significant difference in the selected variables between farmers who use AI and those who use bull service. In the second approach, selected variables (and associated factor scores) that were hypothesized to influence farmers’ breeding choices were tested using a logistic regression model as follows:

$$ {y}_i={\beta}_0+{\beta}_1{x}_1+{\beta}_2{x}_2+{\beta}_3\ {x}_3+{\beta}_4{x}_4+{e}_1 $$

where *y*_*i*_ is a vector of the breeding method adopted by each farmer; *β*_1_, *β*_2_, *β*_3_, and *β*_4_ are vectors of coefficients associated with each explanatory variable category; *x*_1_, *x*_2_, *x*_3_, and *x*_4_ are incidence matrices that link the fixed effects of the explanatory variable categories (farmer, farm characteristics, income, and institutional, respectively) to the response variable; and *e*_1_ is the error term. All analyses were executed using SAS version 9.4 (SAS 2003).

## Results

### Farmer’s characteristics

#### The difference between AI and bull service users on farmer characteristics

**Fig. 3:**
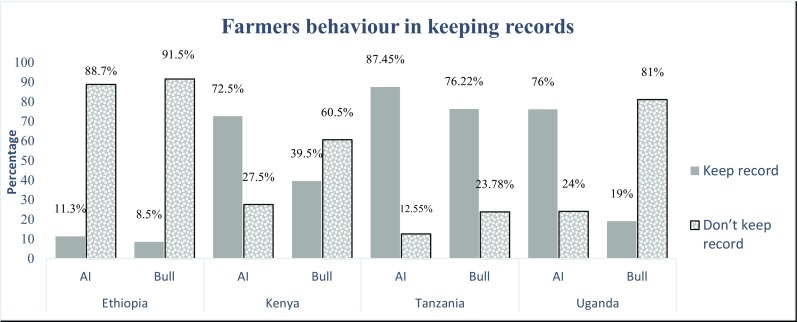
Proportion of farmers who keep records given their choice of breeding method by artificial insemination (AI) and traditional bull mating services

**Fig. 4:**
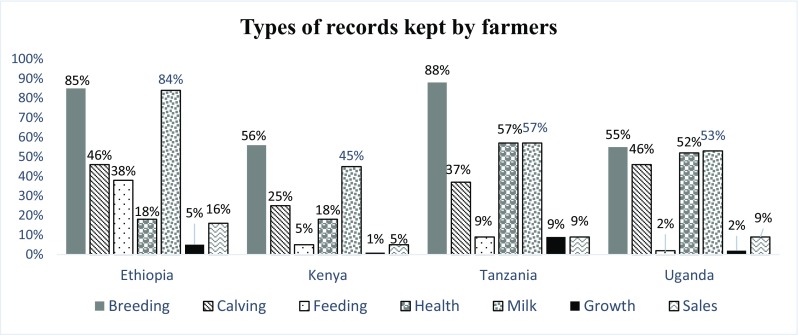
Types of records kept by farmers as a proportion of all records by country

**Fig. 5:**
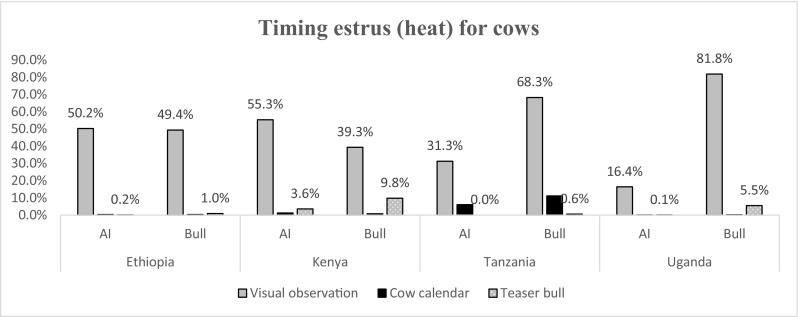
Methods used by farmers to ensure timely estrus (heat) detection in smallholder dairy systems in Eastern Africa

**Fig. 6:**
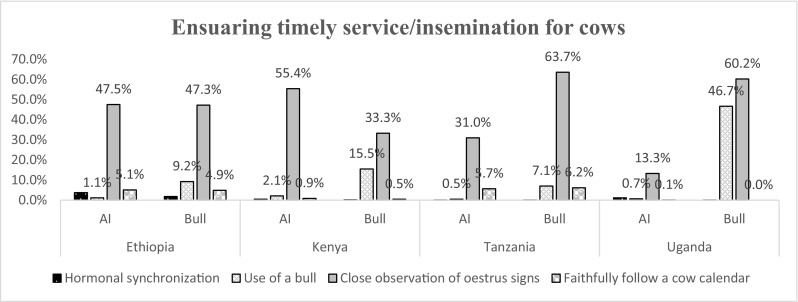
Methods used by smallholder dairy farmers to ensure timely service/insemination in Eastern Africa

**Fig. 7:**
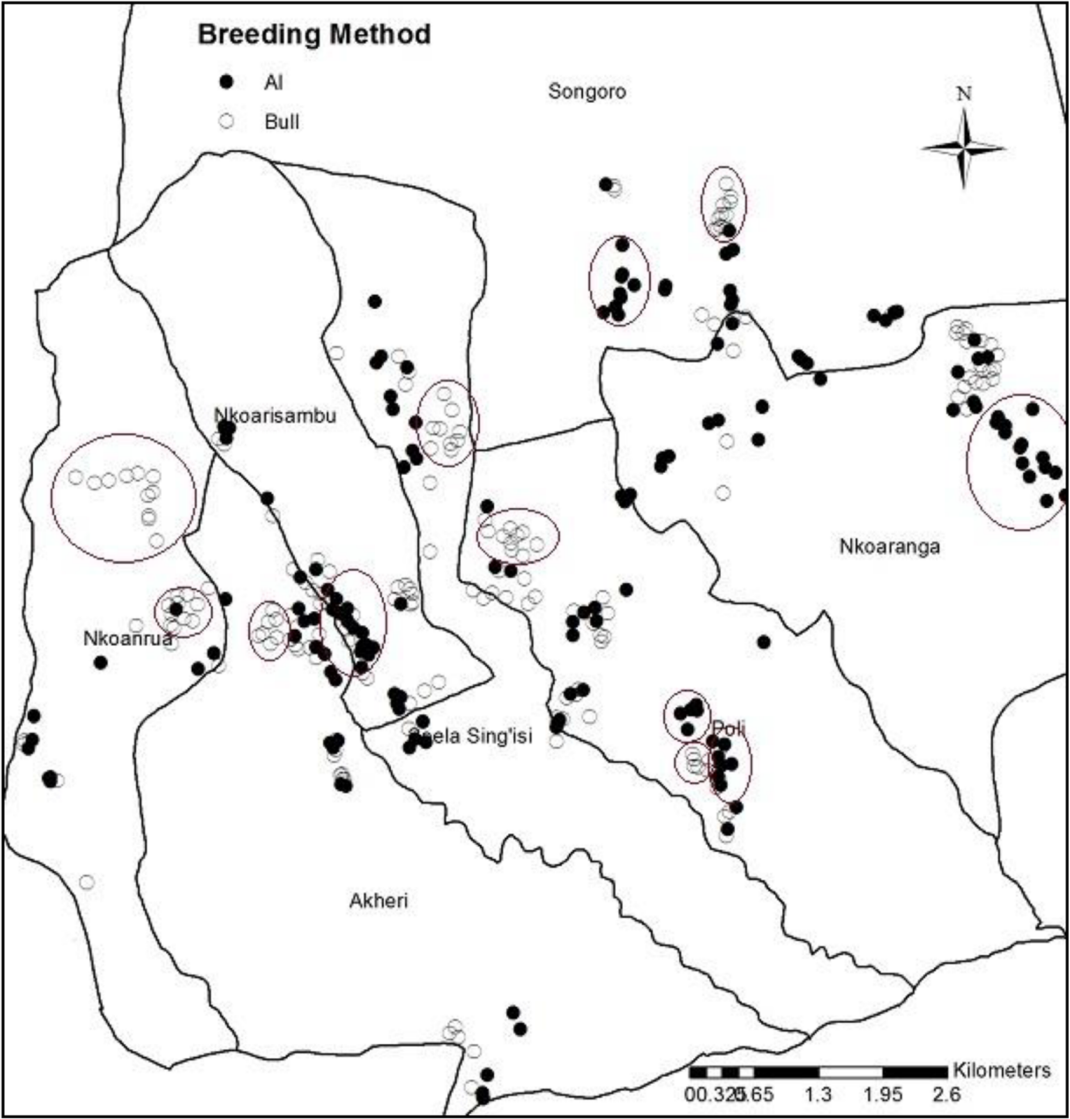
Clusters of farmers based on their breeding method preferences for Arumeru District in Tanzania. The pattern is replicated in all study sites and all countries. Open dots represent farmers who used traditional bull mating while closed dots represent farmers who used artificial insemination

Figures 3, 4, 5, 6 and 7 and Table 2 summarize results obtained on farmer’s characteristics. The average dairy farming experience was similar for both AI and bull service farmers, ranging between 5 and 10 years. Majority of AI farmers (more than 70%) mentioned to kept records in all countries except Ethiopia, where only 12% of AI farmers kept records (Fig. 3). Farmers utilizing AI were more likely to keep records (*P* < .001) compared to those who used bulls in all study countries except for farmers in Ethiopia. The type of records claimed to be kept by farmers were predominantly for animal breeding, health, calving dates, and milk yield (Fig. 4). The majority of farmers (more than 50%) preferred visual observation to other methods for both estrus detection and ensuring timely service/insemination for their cows (Fig. 5 and Fig. 6).

**Table 2:** Differences between farmers who use artificial insemination and those who use traditional bull mating in education level, years of experience in dairying, record keeping practices, and membership to farmer groups. Values are either mean ± SD or proportions

Variable		Ethiopia			Kenya			Tanzania			Uganda		
		AI	Bull	Significant difference	AI	Bull	Significant difference	AI	Bull	Significant difference	AI	Bull	Significant difference
Years in formal education		6.52 ± 5.1	4.49 ± 4.6	< .0001	10.16 ± 4.8	9.80 ± 4.6	0.0089	9.425 ± 3.3	8.26 ± 3.1	< .0001	10.55 ± 6.4	9.48 ± 6.6	0.0042
Years of experience in dairy faming		2.4 ± 1.52	2.19 ± 1.47	< .0001	10.18 ± 2.6	9.80 ± 2.6	0.0103	9.41 ± 1.8	8.29 ± 1	< .0001	10.55 ± 1.7	9.48 ± 1.7	0.09
Belong to farmers groups	No	80.12%	78.21%	0.2338	74.98%	77.12%	0.1000	82.12%	74.21%	< .0001	86.92%	87.11%	0.92
	Yes	19.88%	21.79%		25.02%	22.88%		17.88%	25.79%		13.08%	12.89%	
Keeping record	No	88.68%	91.46%	0.0184	39.54%	72.52%	< .0001	12.55%	23.78%	< .0001	23.84%	80.55%	< .0001
	Yes	11.32%	8.54%		60.46%	27.48%		87.45%	76.22%		76.16%	19.45%	

Additionally, in all study countries, farmers who used AI had on average longer formal schooling years (up to 1 year more) than those who used bull service. Further, irrespective of breeding method utilized, most farmers (80%) did not belong to any farmer groups, even though these groups existed. There was a clear grouping of farmers into spatial clusters as shown in Fig. 7, indicating the influence of neighbors in dairying. These clusters coincided with the preferred method of breeding given that farmers in very close clusters tended to choose similar methods of breeding.

#### Farmer characteristics influencing breeding methods

The farmer’s characteristics associated with choice of breeding method are presented in Table 3. There was a significant relationship between records keeping and the use of AI. Farmers, who used AI, kept animal breeding, health, calving, and milk yield records. This positive relationship between records keeping and AI was observed in Kenya (*p* < 0.001), Tanzania (*p* < 0.001), and Uganda (*p* = 0.004) but not in Ethiopia. While dairy farming experience had a significant impact on choice of AI in Ethiopia (*p* = 0.001) and Tanzania (*p* = 0.003), there was no relationship between AI adoption and the level of education of the particular farmer in all four countries.

**Table 3:** Farmer characteristics and their influence on choice of breeding method

Variables	Ethiopia		Kenya		Tanzania		Uganda	
	Estimate	Pr > chi-Sq	Estimate	Pr > chi-Sq	Estimate	Pr > chi-Sq	Estimate	Pr > chi-Sq
Years in formal education	0.0236	0.1027	− 0.0161	0.3085	− 0.0109	0.6293	− 0.0178	0.4551
Total number of children in the household	− 0.0112	0.6869	− 0.00074	0.9183	0.0956	0.0083	0.0129	0.8262
Keeping record	0.00503	0.9825	0.5774	< .0001	0.9721	< .0001	1.0071	0.004
Experience of head of household in dairy	0.1548	0.0004	0.0312	0.3802	0.1134	0.0038	0.0832	0.3297
Farmer belonging to farmers’ group	− 0.3261	0.0439	0.011	0.9477	− 0.1754	0.2964	0.1123	0.807

### Farm characteristics

#### The difference between AI and bull service users on farm characteristics

Generally, in all countries, farmers preferred two types of feeding systems: grazing and stall feeding. Ethiopia and Tanzania shared the same pattern, where farmers preferred stall feeding (more than 57%) while in Kenya and Uganda, more farmers (more than 40%) preferred grazing to stall feeding (less than 20%). This pattern was observed in both rainy and dry seasons. However, it is important to note that for Kenya, the sites targeted for the study were in one part of the country where land sizes were relatively large, and also, the traditional socio-cultural behaviors favored grazing. Other major dairying regions in Kenya mainly practice stall feeding. There was a marginal effect of seasonality on the chosen feeding system. Generally, increases of between 5 and 8% were observed for stall feeding during the dry season in Ethiopia and Uganda. In all countries, farmers using AI preferred stall feeding to grazing compared to farmers using bulls as shown in Table 4.

**Table 4:** Proportional of farmers utilizing either of grazing or stall feeding, categorized by the type of breeding method they use

Country	AI use (%)	AI user feeding preference				Bull user feeding preference			
		During wet season		During dry season		During wet season		During dry season	
		Stall (%)	Grazing (%)	Stall (%)	Grazing (%)	Stall (%)	Grazing (%)	Stall (%)	Grazing (%)
Ethiopia	50.35	57.3	15.4	64.8	11.9	30.7	8.8	64.8	6.8
Kenya	56.50	10.9	38.4	11.5	40.47	7.6	48.7	7.7	42.10
Tanzania	31.46	94.4	2.06	94.2	2.46	79.44	5.95	79.13	5.95
Uganda	13.46	51.7	20	56.6	20.1	3.8	79.4	3.7	76.6

For Kenya and Uganda, there was no significant difference between AI and bull users in terms of the total land size per farmer. However, significant differences were observed for Ethiopia (*P* < .001) and Tanzania (*P* = 0.03). In Ethiopia, farmers who kept bulls had on average 1.5 acres of land more than those who had adopted AI. In the case of Tanzania, the opposite was true with farmers that used AI having 0.4 acres of land more than bull service users. In terms of average herd size per household, significant differences (*P* < 0.001) were observed between the two groups in Ethiopia and Uganda. Farmers who used bull service tended to have a larger herd size, having on average two more animals than those who used AI. The number of milking cows tended to be equal among both groups and a slight difference for farmers in Uganda where bull service users had one more milking cow than AI users (Table 5).

**Table 5:** Farm characteristics for farmers using either bull mating or artificial insemination. Values are mean ± SD

Variable	Ethiopia (2892)			Kenya (4400)			Tanzania (3236)			Uganda (2555)		
	AI, 1456 (50.35%)	Bull, 1436 (49.65%)	Significant difference (*P* value)	AI, 2486 (56.5%)	Bull, 1914 (43.5%)	Significant difference (*P* value)	AI, 1018 (31.46%)	Bull, 2218 (68.54%)	Significant difference (*P* value)	AI, 344 (13.46%)	Bull, 2211 (86.54%)	Significant difference (*P* value)
Total land size (acres)	5.2 ± 2.3	6.7 ± 3.3	< .0001	6.62 ± 9	6.39 ± 8.9	0.41	3.73 ± 5.6	3.36 ± 4	0.0358	15.07 ± 18.6	16.78 ± 18.6	0.1163
Total livestock number	8 ± 5.06	10 ± 5.6	< .0001	7 ± 4.4	7 ± 4.7	0.65	5 ± 4.7	5 ± 4.7	0.09	11 ± 9	13. ± 9.4	< .0001
No of milking cows	3 ± 1.08	3 ± 0.9	0.4727	2.81 ± 1.2	2.79 ± 1.2	0.65	2 ± 0.7	2 ± 0.6	0.2493	3 ± 1.3	4 ± 2	< .0001
Average milk production at peak for the best animal (L/day)	14.42 ± 6.3	12.73 ± 5.8	< .0001	13.08 ± 4.9	12.15 ± 5.2	< .0001	11.77 ± 0.9	13 ± 0.7	< .0001	15.09 ± 5.4	9.75 ± 3.5	< .0001
Average milk production at peak for the worse animal (L/day)	9.75 ± 4.7	8.54 ± 4.1	< .0001	8.65 ± 3.5	8.24 ± 3.6	< .0001	9.41 ± 4	8.39 ± 3.5	< .0001	8.24 ± 4.1	5.32 ± 2.3	< .0001
Lactation length for the best cow (months)	310 ± 105	309 ± 92	0.8746	331 ± 129	327 ± 126	0.3374	312 ± 64	312 ± 61	0.9816	350 ± 126	262 ± 75	< .0001
Lactation length for the worse cow (months)	258 ± 72	256 ± 66	0.3366	282 ± 97	282 ± 97	0.8946	296 ± 50	293 ± 53	0.0761	245 ± 107	204 ± 75	< .0001
Average working hours per workers (h)	2 ± 3.4	1.8 ± 3.3	0.18	2.2 ± 3.4	1.8 ± 3.1	< .0001	2.2 ± 3.4	1.62 ± 3.3	< .0001	6.1 ± 4.08	4.3 ± 4.4	< .0001
Average monthly wage per worker	174.7 ± 322	136.1 ± 472	0.01	904.4 ± 1673	682.5 ± 1463.2	< .0001	18,736.8 ± 32,979	10,463.7 ± 25,654	< .0001	75,000 ± 54,832	40,000 ± 42,226	< .0001

In general, farmers who used AI had higher milk yields compared to those who used bull service. There was a significant difference (*P* < .001) in peak milk yield between the two groups in all countries except for Tanzania. On average, cows of farmers who used AI yielded more (14.42 ± 6.3 L) during peak lactation than cows from farms using bulls (12.73 ± 5.8 L). In Kenya, cows from farmers who used AI yielded 13.08 ± 4.9 L at peak compared to those from bull bred farms which yielded 12.15 ± 5.2 L at peak. In Contrast, the trends in Tanzania were reversed, with cows from AI farmers yielding lower at peak than cows from bull breeding farms, at 11.77 ± 0.9 L and 13 ± 0.7 L, respectively. In Uganda, farmers who used AI averaged 15.09 ± 5.4 L at peak while those who used bulls had peak yields almost 45% lower at 8.24 ± 4.1 L. Additionally, for farmers in Uganda, there was a huge difference (approximately 90 days) in cow lactation length between the two groups (*P* < 0.001), with farmers who used AI service experiencing longer lactation periods than those who used bulls. There were no significant differences between the two groups in lactation lengths for the other countries (Table 7).

In addition, farmers who used AI engaged their workers for longer periods, on average. Their workers put in between 3 to 7 h/day while workers in bull service farms worked 1 to 4 h/day. Farmers who used AI paid their workers more than those who used bull service.

#### Farm characteristics influencing breeding methods

A number of farm-related factors were evaluated for their effect on AI adoption as shown in Table 6. Results indicate a strong inverse relationship between AI use and herd sizes in Ethiopia and Uganda. Farmers with large herd sizes tended to use bull mating while small farms used AI. The number of months for fodder purchases had a significant relationship with AI use in Ethiopia, Kenya, and Tanzania. Husbandry practices such as frequency of watering had a positive (Ethiopia) and negative (Tanzania) relationship with AI usage. Similarly, the frequency of deworming and treatment showed a positive relationship with AI usage in Tanzania.

**Table 6:** Farm characteristics and their influence on choice of breeding method

Variables	Ethiopia		Kenya		Tanzania		Uganda	
	Estimate	Pr > chi-Sq	Estimate	Pr > chi-Sq	Estimate	Pr > chi-Sq	Estimate	Pr > chi-Sq
Total land size	− 0.084	0.0238	0.00157	0.8671	0.0309	0.1675	0.0142	0.0662
Total livestock number	− 0.0224	0.1317	0.00696	0.6874	0.0411	0.2479	− 0.0246	0.1489
Number of months purchasing fodder	− 0.1029	< .0001	0.12	0.0032	0.0635	0.0015	− 0.0107	0.9113
Number of months purchasing crop-residue	0.00792	0.6555	0.000572	0.9782	0.0901	0.0004	0.0481	0.1304
Number of months purchasing concentrate	0.0178	0.464	0.0296	0.1186	0.0784	< .0001	− 0.0504	0.3622
Total number of workers in the household	0.061	0.3306	0.0327	0.5994	0.4059	0.0798	− 0.1652	0.2112
Average monthly wage per worker	− 0.1268	0.0767	− 0.0542	0.3345	− 0.0508	0.4142	0.00568	0.9399
Frequency of watering animals	0.3575	0.0003	− 0.0841	0.3093	− 0.3958	< .0001	0.1818	0.3005
Frequency of vaccinating animals	− 0.065	0.6061	0.0604	0.7117	− 0.2546	0.2083	− 0.3503	0.7386
Frequency of deworming animals	0.052	0.3305	− 0.127	0.0837	0.2026	0.0167	0.0103	0.9465
Frequency of treating animals	− 0.0168	0.534	− 0.0639	0.3953	0.1068	0.0241	0.2398	0.0889
Factor 1 (milk production)	− 0.1506	0.039	− 0.0144	0.8714	0.0916	0.2928	0.3752	0.0192
Factor 2 (lactation length)	− 0.0625	0.3428	− 0.048	0.5168	− 0.039	0.5863	0.1424	0.3517

### Institutional settings

#### The difference between AI and bull service users on institution settings

Table 7 summarizes the results obtained on institution setting of the farm. Results indicate that access to the preferred service was higher for AI than for bull service. This was true for all surveyed countries with more than 88% of AI service users being able to access the AI service: (Ethiopia, AI = 86.33%; bull = 56.3%), (Kenya, AI = 88.21%; bull = 64.7%), (Tanzania, AI = 94.99; bull = 62.13%), and (Uganda, AI = 99.42%; bull = 63.13%).

**Table 7:** Parameter estimates for institutional setting variables for farms that use either bull mating or artificial insemination in Eastern Africa. Values are Mean ± SD or proportions

		Ethiopia			Kenya			Tanzania			Uganda		
Variable		AI	Bull	Significant 4difference	AI	Bull	Significant difference	AI	Bull	Significant difference	AI	Bull	Significant difference
Spend per week on purchasing water		10.6 ± 20.9	5.2 ± 15.3	< .0001	7.8 ± 48.1	7.9 ± 54.3	0.93	900.5 ± 1844	505 ± 1598	< .0001	3967.21 ± 19,273	1295.86 ± 23,849	0.0213
Distance to the watering point (Km)		0.46 ± 0.9	0.69 ± 0.9	< .0001	0.26 ± 0.52	0.29 ± 0.57	0.026	0.174 ± 0.4	0.4 ± 0.8	< .0001	0.21 ± 0.33	0.4436 ± 0.61	< .0001
Cost of transport to milk buyer		0.5 ± 2.6	0.29 ± 1.8	0.0063	10.29 ± 64	9.58 ± 27	0.63	160.1 ± 517	212.0 ± 604	0.019	264.1 ± 780	129.5 ± 568	0.0022
The average travel time on foot to buyer (h)		0.14 ± 0.4	0.16 ± 0.4	0.1987	0.50 ± 1	0.5 ± 0.92	0.82	0.23 ± 0.5	0.30 ± 0.78	0.012	0.26 ± 0.7	0.2 ± 0.5	0.07
Times visited by extension officer		2	1	0.0003	1	1	0.66	11	11	0.8591	2	2	0.4308
Distance to market		3.26 ± 2.5	4.06 ± 2.6	< .0001	3.52 ± 2.3	3.61 ± 2.2	0.23	2.8016 ± 2.2	2.53 ± 2.3	0.0020	2.9 ± 2.2	3.49 ± 2.3	< .0001
Cost per breeding service		28 ± 40.5	24 ± 38.8	0.0300	1286.9 ± 581	900.4 ± 582	< .0001	12,925 ± 3781	8016.3 ± 5987	< .0001	61,752.9 ± 27,952	27,170.5 ± 35,736	< .0001
Distance to service provider		2.3 ± 2.8	1.4 ± 2.4	< .0001	4.2 ± 3.5	3.4 ± 3.3	< .0001	3.19 ± 3.01	1.05 ± 1.2	< .0001	7.30 ± 7.4	4.64 ± 7.4	< .0001
Find preferred breeding method	No	199 (13.67%)	628 (43.7%)	< .0001	293 (11.79%)	676 (35.3%)	< .0001	51 (5.01%)	840 (37.87%)	< .0001	2 (0.58%)	815 (36.87)	< .0001
	Yes	1257 (86.33%)	808 (56.3%)		2193 (88.21%)	1238 (64.7%)		967 (94.99%)	1378 (62.13%)		342 (99.42%)	1396 (63.13%)	

In terms of breeding service fees, AI users paid double the price paid by bull users (*p* < 0.001) and their service providers traveled long distances (*p* < 0.001) to provide the service. However, farmers who used bull service had to travel long distances (*p* < 0.001) of up to 2 Km more, to access water and market services. With regard to extension services and capacity development, very few farmers (24%) had attended any training session within the year of the study. This illustrates the scarcity of extension agents and services.

#### Institutional settings influencing breeding methods

Table 8 presents the results obtained on institutional setting. Factors, such as the breeding method that led to recently calved, the number of times a farmer had used AI (frequency of using AI), and accessibility to breeding method, all had positive significant association to the breeding decision the farmer adopted in all countries. The number of services before conception was negatively associated with choice of AI as a breeding method in Ethiopia, Tanzania, and Uganda. Distance to the service provider had a negative correlation with AI use in Ethiopia and Tanzania. In all study countries, cost of service was associated with the choice of breeding method.

**Table 8:** Estimates for variables with a significant association with choice of breeding method in Eastern Africa

	Ethiopia		Kenya		Tanzania		Uganda	
	Estimate	Pr > chi-Sq	Estimate	Pr > chi-Sq	Estimate	Pr > chi-Sq	Estimate	Pr > chi-Sq
Number of times have used AI	0.5423	< .0001	1.4712	< .0001	0.6472	< .0001	0.9778	< .0001
Breeding method recently calved	3.9986	< .0001	4.3308	< .0001	4.805	< .0001	4.1653	< .0001
Number of AI or bull before conception	0.2078	0.0231	− 0.1669	0.1565	− 0.2923	0.0195	− 0.3786	0.0491
Find preferred breeding method	0.6584	< .0001	0.8097	< .0001	0.6557	0.0012	0.3971	0.6065
Average cost per breeding service	0.2284	0.0245	1.1171	< .0001	0.8559	< .0001	0.8045	0.0004
Distance to service provider	0.0321	0.0225	− 0.00043	0.8511	0.4358	< .0001	− 0.00388	0.8347
Distance to the watering point (Km)	− 0.00481	0.9583	− 0.0802	0.2531	− 0.4828	0.001	0.00162	0.9301
Amount spend per week on purchasing water	0.1039	0.3856	− 0.1874	0.2326	− 0.0969	0.0447	− 0.0442	0.7367
Distance to buyer	− 0.0222	0.6264	0.00665	0.7264	− 0.00428	0.9007	− 7.93 E − 06	0.9996
Cost of transport to\buyers	0.3173	0.4024	− 0.1569	0.2734	0.0896	0.2229	0.1121	0.5069
Times visited by extension officer	0.0316	0.1311	0.1435	0.0311	0.000245	0.4416	− 0.0298	0.6791
Distance to market	0.0199	0.4867	0.0646	0.0281	0.0571	0.1053	− 0.044	0.5183
Availability of vaccination service	0.00942	0.9696	− 0.0229	0.9327	0.3161	0.2761	0.6314	0.5981
Availability of water	0.3205	0.0803	− 0.0399	0.8022	0.3724	0.1745	− 0.1409	0.723

Only in Tanzania was the amount of money a farmer spent to purchase water and the distance traveled to the watering point negatively associated with the farmer’s breeding choices. Water availability and choice of breeding method had a positive association in Ethiopia but a negative one in Kenya and Uganda. The cost of transporting milk to market and the distance traveled to these markets had no relationship with choice of breeding method. Given the negative correlation coefficient despite lack of significance, there is a trend to suggest a negative effect of distance to market on AI choice.

### Farm income

#### The impact of using AI and bull services on farm income

Table 9 presents the results on farm income. Comparing the total amount of milk sold per day by a farm, farmers who used AI sold more milk than those who used bull service in Ethiopia, Kenya, and Tanzania (difference of 3 L, *P* < 0.001), Kenya (difference of 2 L, *P* = 0.01), and Tanzania (difference of 2 L, *P* = < 0.001). However, there was no significant difference between the two groups except in Tanzania in the amount of income obtained from selling the product.

**Table 9:** Differences in household income for farmers using AI or bull mating as a breeding option. Values are Mean ± SD

Variable	Ethiopia			Kenya			Tanzania			Uganda		
	AI	Bull	Significant difference	AI	Bull	Significant difference	AI	Bull	Significant difference	AI	Bull	Significant difference
Average liters sold	12.69 ± 14.4	9.66 ± 11.3	< .0001	10 ± 9	8 ± 9	< .0001	10.27 ± 10	8.0 ± 8.2	< .0001	13.09 ± 14	13.28 ± 14	0.8030
Total income from crops (′000)	440.4 ± 210.5 (ETB)	403.6 ± 252.47 (ETB)	0.6703	205.4 ± 289.5 (Kshs)	198.4 ± 134.7 (Kshs)	0.79	520.6 ± 126.7 (Tshs)	429.2 ± 110.3(Tshs)	< .0001	634.8 ± 177.1 (UGX)	611.5 ± 164.9 (UGX)	0.1221

#### Farm income influencing breeding methods

Income from setting dairy products had a positive relationship with the use of AI in Ethiopia but had a negative association in Tanzania, Uganda, and Kenya.

## Discussion

Despite the benefits of AI in enhancing herd productivity, the method/technology has had a low adoption rate. With respect to AI, in order to formulate relevant breeding policies, there is a need to understand the drivers of producer’s preference for a particular breeding service. This study was undertaken first to evaluate if there were differences between farmers who used AI and those who used bull service with regard to animal husbandry practices and infrastructure settings. The second objective was to identify factors that influence breeding decisions in Eastern Africa and to assess if investigated factors cut across the regions. This study contributes to the livestock sector on breeding practices and general animal husbandry practices for small-scale dairy farmers. Compared to other studies, this study involved more number of farmers and covered a wide area of sub-Saharan Africa: Ethiopia, Kenya, Tanzania, and Uganda.

### The difference between AI and bull users

Generally, majority of farmers in the surveyed countries did not keep track of what was happening on their farms by way of performance records. These findings are broadly consistent with what was observed in the case study on smallholder farmers’ willingness to adopt dairy performance recording in Malawi and Rwanda (Chagunda et al. 2006; Chatikobo et al. 2009). In Chagunda et al.’s (2006) study, farmers with very small (less than two animals) or large herd sizes were associated with low recording, while farmers with three to six cows showed the highest propensity to keep records. In our study, the herd size for the surveyed households ranged between five and 13 animals. This poses a challenge to farmers because the recording exercise for many animals can be cumbersome, and as a result, farmers choose not to keep records.

The significant differences observed between farmers who used bulls vs. AI service with regard to record keeping has been reported before. Nishimwe et al. (2015) reported that record keeping was a necessary routine practice for AI users because of the nature of tracking required to identify successful conception. In that study, a significant relationship between keeping of records and pregnancy rates of animals, with farmers who keep records experiencing higher animal pregnancy rates compared to those who do not keep records, is observed. The nature of AI and the type of records required (time and date of heat detection, date of insemination, and date of repeat insemination) means that records related to breeding and animal reproduction would dominate the type of records kept by farmers. It is interesting to note that even though majority of farmers indicated that they keep breeding records, these same farmers did not use any animal calendar to track the time to breed. This poses a question on whether farmers really use these records for decision-making and improvement of reproductive efficiency.

Farmers’ preferences to keep only certain type of records signify that farmers are not aware of the importance of keeping other critical sets of records. Successful farm management requires having a good, useful set of farm records, such as records on breeding, production, sales, farm expenses, and animal feeding. These records do not ensure that the farm will be productive, but success of these farms is unlikely without them. Yet, the majority of respondent who keep records indicated that they use those records for self-evaluation and management practices. The implication of farmers not keeping sales records can be considered as a red flag towards describing a farm’s business performance. It is recommended that when a farmer keeps all sets of data for a specific time, he/she will be able to compare all expenses incurred in relation to the amount of milk produced. Further, keeping accurate facts and figures on the business performance helps a farmer with access to funds from banks and when seeking government support. However, the current study did not investigate how farmers extract information from collected records. In this regard, there is a need to investigate how farmers use their records for decision-making.

Two types of feeding systems were dominant: grazing and stall feeding. One implication of farmers choosing stall feeding over grazing was the cost incurred to purchase water. Most farmers who adopted grazing watered their animals from ponds, rivers, and other similar but “free” water sources during grazing. On the other hand, farmers who adopted stall feeding spent more money to purchase water because water had to be brought to the animals, and this is often not available free of charge. Baltenweck et al. (2004) observed that farmers who use natural service (bull) tend to apply extensive production systems based mainly on grazing in contrast to those who use AI that apply intensive production systems (mainly or only stall feeding).

### Factors influencing adoption of AI as breeding method

Natural service tended to be the most preferred breeding method on surveyed sites in Tanzania and Uganda. Ethiopia maintained a similar distribution: 50% of AI users and 50% of bull users. Kenya has more number of AI users than bull users. The main reason that natural service is the most preferred in Uganda and Tanzania is because farmers cannot easily access semen where they are. For the case of Tanzania, it was noted that most of farmers who use AI were located in the northern part of the country, where the National Artificial Insemination Centre (NAIC) is located compared to the southern part where farmers depend on semen transported from the AI unit and other smaller distribution points. This system is not working perfectly, and availability of semen is not guaranteed. Moreover, the case for Uganda having more number of farmers who use bull is an indication that farmers prefer bulls than AI. The case remained to be the same as it was reported by the Office of the Auditor General in Uganda (2015) that there has been a considerable decline in the use of AI service due to poor access occasioned by dependency on a single supplier, the NAGRIC AI unit in Entebbe.

Farmers in Uganda were favored by demographic factors. For instance, farmers had farm sizes three to five times bigger than those owned by farmers in the other surveyed countries. This allows for relatively easy access to animal feed and large areas to rear bigger herds, hence the preference for bulls. Also, it is worth noting that the predominant use of bull service in Uganda probably resulted into remarkably shorter lactation lengths, symbolizing shorter calving intervals compared to the other countries. The use of bulls ensures that animals on heat are readily served when in estrus as opposed to AI service which may delay because a service provider is not available, or heat is detected late. The numbers of services before conception were found to negatively affect adoption of AI. This was the case in all countries. These findings are consistent with those by Anthony et al. (2014) who reported that farmers who use bulls had low numbers of repeats. Also, in the event that the service was not successful, there were no extra charges levied for repeating insemination (Anthony et al. 2014). This is not usually the case for AI users.

Moreover, poor heat detection can result in low conception rate (low fertility estimates). Although results from the current study indicated that the majority of farmers visually observed for heat in cows, this method alone is not efficient. This case was also observed by Harlow et al. (2015) who reported that there were high incidences for farmers missing the opportunity to either inseminate or mate their animals when they use visual inspection. This is because farmers in mixed crop-livestock systems, which is common to small-scale farmers, tend to spend less time in the cow sheds and are therefore less likely to see cows in estrus. However, the current study did not investigate the reasons for their preference of visual inspection as opposed to other methods available to them.

Despite the fact that the cost of service was associated with choice of breeding service, the impact of cost on enhancing a farmer on AI adoption was very minimum. This indicates that farmers were willing to pay any reasonable cost in order to get AI service. The results further showed that farmers who use AI paid more than those who used bulls. Regardless of challenges faced by farmers who used AI, it was important to note that farmers continued using the AI service in subsequent inseminations. The more the farmers continued using AI, the more the proportion of farmers adopting the technology increased. This implies that once farmers have started using the service, they do not stop as long as the service is available. The same results were observed in Rwanda where despite the fact that the artificial insemination service had many constraints, its acceptability was very high (Makuza et al. 2016).

The probability of using AI was related to the proportion of farmers buying animal feeds, with farmers who bought AI service also bought animal feeds. This is similar to what was reported by Tefera et al. (2014), who indicated a positive relationship between supplementation of purchased feed with the adoption of AI technology. Also, Nalunkuuma et al. (2015) reported that farmers who practiced zero grazing, which is associated with buying of feeds, were keen on improving reproduction of their animals.

Farmer education level was not associated with adoption of AI. This could be that informal knowledge sharing among farmers as well as experience gained through actual practice provided the vital information to the farmers more than formal education would. Utilizing the existing clusters of farmers using the same type of breeding technology may be an effective way of implementing knowledge exchange programs. It may be that the best way to increase adoption of AI and related technologies is to use established peer-to-peer knowledge transfer mechanisms. This is best exemplified in the clusters of neighbors opting for similar breeding options as seen in this study.

Results in the current study indicate that there is a need to improve access to quality breeding services, both in terms of AI services and appropriate breeding bulls. If adoption of AI is seen as the more desirable option given its obvious advantages and portability, it is imperative that the requisite systems be put in place to ensure wider, easier, and faster access where needed. However, in ensuring that the system works efficiently, it is important to ensure that appropriate infrastructures and tools are available to farmers when and where they are needed. Use of tools such as cow-side heat detection methods can go a long way in improving the delivery of AI services. Also, because heat detection methods are supported with visual observation, farmers need to be trained on how best to track days to estrus onset and ensure timely detection of the estrus to ensure the service providers are available within the recommended breeding window.

This study has demonstrated, from the farmers perspective, the need to improve supporting infrastructure and services such as water accessibility, provision of appropriate animal feed, and animal health service. As these factors were found to affect the efficiency of farmer to adopt the best husbandry practices, which in turn, affect the adoption of AI. To this end, revamping of extension services to strengthen farmer training, capacity building, and knowledge exchange is critical.
